# Neuronal α_2_δ proteins and brain disorders

**DOI:** 10.1007/s00424-020-02420-2

**Published:** 2020-06-30

**Authors:** Cornelia Ablinger, Stefanie M. Geisler, Ruslan I. Stanika, Christian T. Klein, Gerald J. Obermair

**Affiliations:** 1 Institute of Physiology, Medical University Innsbruck, 6020 Innsbruck, Austria; 2 Department of Pharmacology and Toxicology, University of Innsbruck, 6020 Innsbruck, Austria; 3 Division Physiology, Karl Landsteiner University of Health Sciences, 3500 Krems, Austria; 4 Department of Life Sciences, IMC University of Applied Sciences, 3500 Krems, Austria

**Keywords:** Neurological disease, Autism, Schizophrenia, Epilepsy, Synapses, CACNA2D1–4

## Abstract

α_2_δ proteins are membrane-anchored extracellular glycoproteins which are abundantly expressed in the brain and the peripheral nervous system. They serve as regulatory subunits of voltage-gated calcium channels and, particularly in nerve cells, regulate presynaptic and postsynaptic functions independently from their role as channel subunits. α_2_δ proteins are the targets of the widely prescribed anti-epileptic and anti-allodynic drugs gabapentin and pregabalin, particularly for the treatment of neuropathic pain conditions. Recently, the human genes (CACNA2D1–4) encoding for the four known α_2_δ proteins (isoforms α_2_δ-1 to α_2_δ-4) have been linked to a large variety of neurological and neuropsychiatric disorders including epilepsy, autism spectrum disorders, bipolar disorders, schizophrenia, and depressive disorders. Here, we provide an overview of the hitherto identified disease associations of all known α_2_δ genes, hypothesize on the pathophysiological mechanisms considering their known physiological roles, and discuss the most immanent future research questions. Elucidating their specific physiological and pathophysiological mechanisms may open the way for developing entirely novel therapeutic paradigms for treating brain disorders.

## Introduction

α_2_δ proteins are membrane-anchored extracellular glycoproteins which have initially been identified as subunits of voltage-gated calcium channels (VGCCs). An increasing number of studies, however, suggest functions independent of the calcium channel complex. α_2_δ proteins are the targets of the widely prescribed anti-epileptic and anti-allodynic drugs gabapentin and pregabalin and have been linked to a large variety of diseases. In this review, we provide an overview of the known and putative associations of α_2_δ genes and proteins with human neurological disorders. Furthermore, we discuss channel-dependent and channel-independent mechanisms of potential relevance for the respective pathophysiological mechanisms.

### α_2_δ genes and proteins

**Fig. 1:**
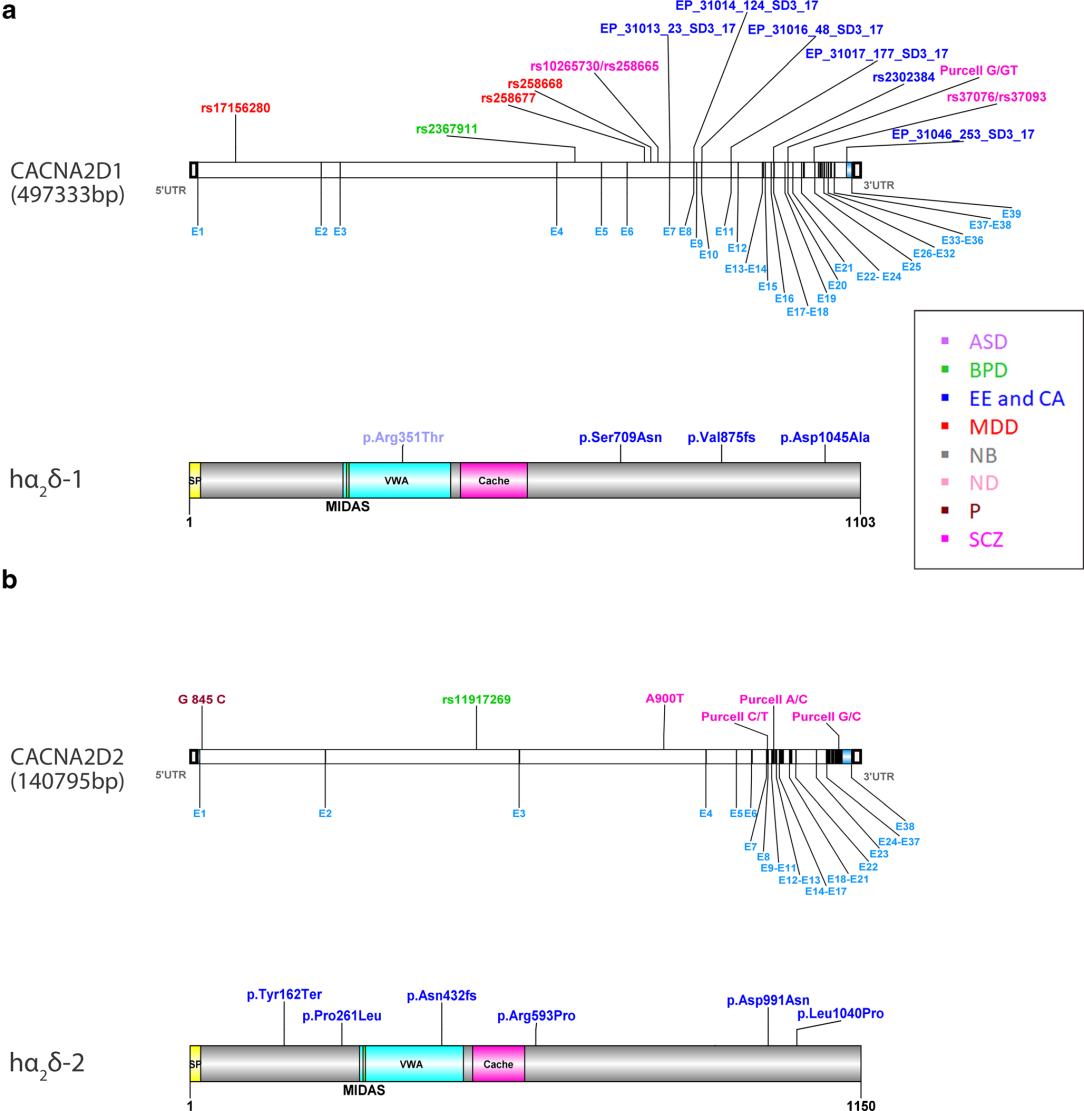
Reference maps of predicted human α_2_δ-1 and α_2_δ-2 neuronal disease mutations and SNPs. Models of CACNA2D1 and CACNA2D2 genes (upper panels in **a** and **b**) and protein structures (lower panels in **a** and **b**), including exon positions (light blue) and protein domains, are based on Ensembl and UniProt databases (ENST00000356253.9/P54289; ENST00000266039.7/Q9NY47). Previously published potentially disease-associated SNPs and disease mutations are indicated (see text for references). The protein structure of all α_2_δ proteins is highly conserved sharing an N-terminal signal peptide (SP, yellow), a von Willebrand factor A domain (VWA, turquois), a cache domain (magenta), and a MIDAS site (green) (see also Fig. 2). ASD (violet), autism spectrum disorders; BPD (green), bipolar disorder; CA (blue), cerebellar atrophy; EE (blue), epileptic encephalopathy; MDD (red), major depressive disorder; NB (gray), night blindness; ND (nude), nicotine dependence; P (bordeaux), pain; SCZ (magenta), schizophrenia

**Fig. 2:**
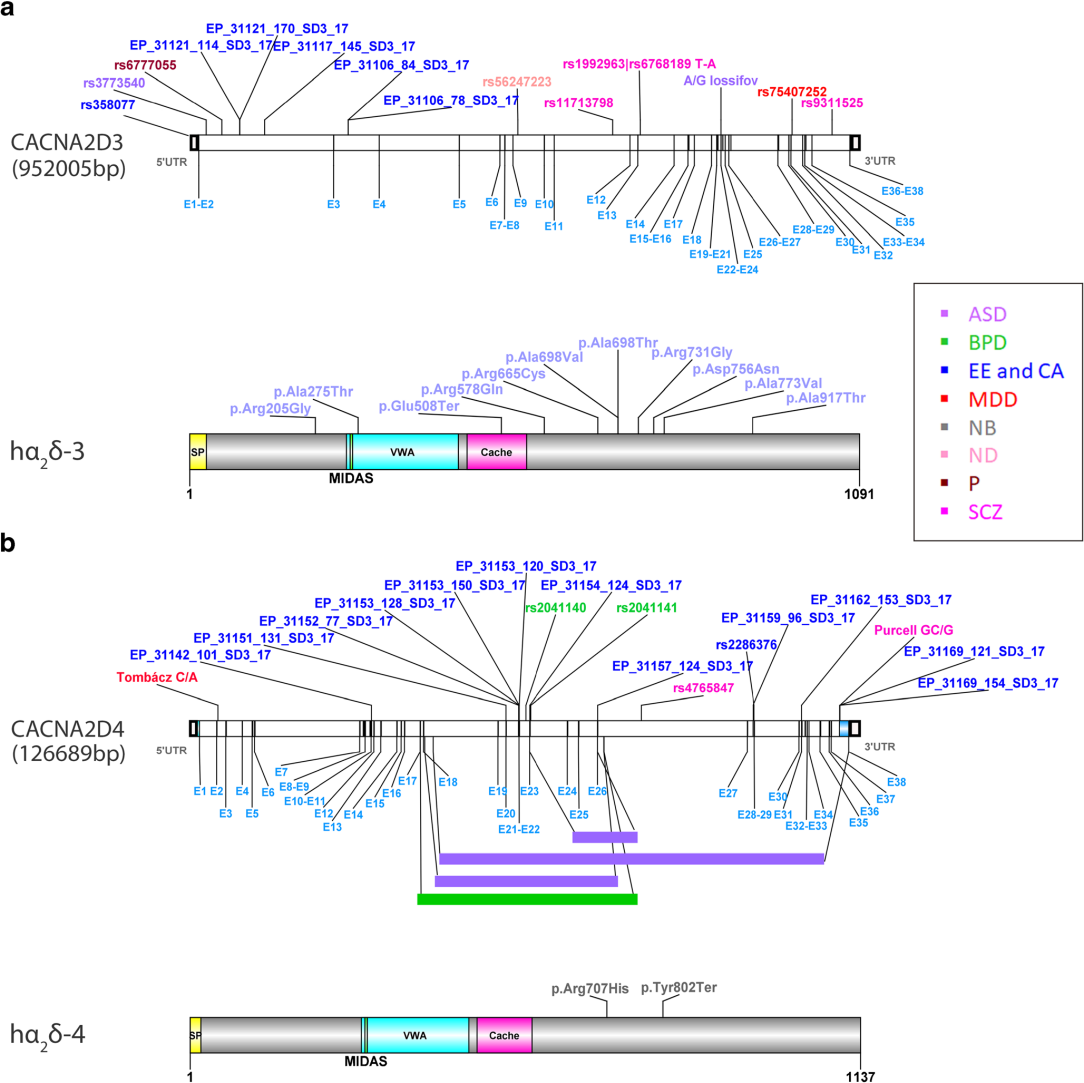
Reference maps of predicted human α_2_δ-3 and α_2_δ-4 neuronal disease mutations and SNPs. Models of CACNA2D3 and CACNA2D4 genes (upper panels in **a** and **b**) and protein structures (lower panels in **a** and **b**), including exon positions (light blue) and protein domains, are based on Ensembl and UniProt databases (ENST00000288197.9/Q8IZS8; ENST00000382722.10/Q7Z3S7). Previously published potentially disease-associated SNPs and disease mutations are indicated (see text for references). Large genomic deletions of CACNA2D4 (**b**) including an inactive cache domain have been linked to ASD (violet bars) and BPD (green bars). α_2_δ-4 protein mutations are so far only known to cause night blindness (NB) and gliomas (not indicated). ASD (violet), autism spectrum disorders; BPD (green), bipolar disorder; CA (blue), cerebellar atrophy; EE (blue), epileptic encephalopathy; MDD (red), major depressive disorder; NB (gray), night blindness; ND (nude), nicotine dependence; P (bordeaux), pain; SCZ (magenta), schizophrenia

In the human genome, four genes (CACNA2D1–4) code for four α_2_δ proteins (α_2_δ-1 to α_2_δ-4), which give rise to multiple transcripts as the result of alternative splicing [31, 60]. The genes contain 38 or 39 exons, and the distribution of exons over the gene is similar between α_2_δ-1 and α_2_δ-2 (Fig. 1) but considerably different in α_2_δ-3 and α_2_δ-4 (Fig. 2). Mature α_2_δ proteins lack the co-translationally cleaved N-terminal signal peptide and are highly glycosylated extracellular proteins of 140 to 170 kDa. They are posttranslationally cleaved into α_2_ and δ moieties, which are covalently bound through disulfide bonds [13, 27, 126]. α_2_δ proteins are most likely attached to the plasma membrane via glycosylphosphatidylinositol (GPI) anchors [26]; alternatively, however, the δ polypeptide chain may form a transmembrane α-helix [91]. Even though all α_2_δ subunits contain well-known protein domains including a von Willebrand factor A (VWA) domain, the detailed structure is still under debate. As of today, the most precise estimation of α_2_δ protein structure is based on a cryogenic electron microscopy (cryo-EM) study of the α_2_δ-1 isoform complexed with the skeletal muscle calcium channel [126]. α_2_δ proteins undergo multiple posttranslational modifications, which make them rather unique, considering that proteolytic processing is a rare feature in VWA-containing proteins. Generally, VWA domains by means of their metal ion–dependent adhesion site (MIDAS) are involved in protein-protein interactions such as extracellular matrix-cell adhesion proteins [124]. While all α_2_δ subunits contain a MIDAS motif, only α_2_δ-1 and α_2_δ-2 incorporate the “perfect” MIDAS motif in which the presence of all five coordinating amino acids is predicted [14, 124]. This implies that structural rearrangement of the protein complex may occur upon divalent cation–dependent complex formation with a protein ligand [31]. Moreover, the MIDAS site is involved in protein-protein interactions that are required to promote the anterograde transport of calcium channels [14, 48]. A perfectly conserved MIDAS motif is not required for metal ion binding [112]; however, it is not yet known whether differences in the MIDAS motif between α_2_δ subunits might account for functional heterogeneity. A role of α_2_δ in extracellular protein interactions is further supported by the presence of cache domains, putative protein interaction sites with homology to the extracellular domains of bacterial chemo-sensing proteins [2, 31].

### α_2_δ proteins as subunits of voltage-gated calcium channels

VGCCs are critical components of all types of excitable cells. They translate membrane depolarization into cellular functions like skeletal and cardiac muscle contraction, nerve cell signaling, hormone secretion, as well as the regulation of gene transcription. In the central nervous system, calcium channels are particularly involved in presynaptic neurotransmitter release and postsynaptic signaling. VGCCs are classified into three low-voltage-gated (Ca_V_3.1–3.3) and seven high-voltage-gated (Ca_V_1.1–1.4, Ca_V_2.1–2.3) channels. High-VGCCs constitute protein complexes classically consisting of a main pore-forming α_1_ subunit and auxiliary intracellular β (β_1–4_) and extracellular membrane–attached α_2_δ (α_2_δ-1 to α_2_δ-4) subunits. While the biophysical channel properties are defined by α_1_ subunits, β subunits regulate membrane expression and other modulatory functions (reviewed in [11]). A wealth of heterologous co-expression studies has illustrated the roles of α_2_δ proteins as calcium channel subunits. Depending on the co-expressed α_1_ subunit, α_2_δ subunits enhance current densities, modulate activation and inactivation kinetics, and can account for a hyperpolarizing shift in the voltage dependence of activation and inactivation (reviewed in [31, 56, 75]). The main proposed mechanism underlying the α_2_δ-induced increase in maximum current is an enhanced plasma membrane expression of channel complexes coupled with a decrease in their turnover [31]. Although it is still elusive how α_2_δ subunits implement their effects, the MIDAS motif seems to be essential, since a MIDAS mutant of α_2_δ-2 causes intracellular retention of α_1_ subunits [14]. Due to their extracellular localization, α_2_δ subunits may also link extracellular signaling entities directly to the calcium channel complex.

### Calcium channel–independent roles of α_2_δ proteins

An increasing number of recent studies suggests that individual α_2_δ isoforms exert specific neuronal functions beyond their classical role as calcium channel subunits. Hence, the affinity and strength of the interaction between α_1_ and α_2_δ are under debate (see discussion in [75]). For example, a comprehensive quantitative study of the neuronal Ca_V_2 channel proteome revealed molar ratios of 0.1–1% of α_2_δ compared to α_1_ and β subunits [71], suggesting a relatively weak and possibly dynamic association. These findings are further corroborated by single-molecule tracking displaying that α_1_ and α_2_δ are only transiently confined together at the cell surface of hippocampal neurons [10, 96, 119], and that α_2_δ-2 accumulation in lipid rafts may be partly independent from its interaction with presynaptic calcium channels [24]. Recent studies particularly implicate α_2_δ proteins as important regulators of synaptic functions and synapse formation, and these functions may be partly or entirely independent of the calcium channel complex (e.g. [18, 35, 40, 59, 97]).

## α_2_δ proteins and neurological disorders

Dysfunctions of voltage-gated calcium channels have been linked to a variety of neurological disorders including Parkinson’s disease, epilepsy, migraine, ataxia, neuropathic pain, and psychiatric disorders [3, 8, 17, 43, 80, 89, 105, 107, 132]. Hence, it is not surprising that likewise α_2_δ proteins have been associated with many of these diseases. The link to neurological disorders is further strengthened by the existence of a variety of knockout and mutant mouse models, which partly represent pathological features of identified human diseases (reviewed in [39]). In the last years, various genome-wide association studies (GWAS) as well as exome sequencing studies have contributed to identifying risk genes for neurological disorders and disease mutations. A further effort in detecting potential disease related genes has been made by single nucleotide variant (SNV) analysis and single nucleotide polymorphism (SNP) genotyping. Along these lines, copy number variation (CNV) analysis contributed largely to the understanding of disease predisposition. SNPs are point mutations of both coding and noncoding DNA regions that occur at a frequency of 0.5–1% in the population. SNV refers to a variation of a single nucleotide mostly arising in somatic cells. Both SNPs and SNVs can affect gene transcription or may generate splice sites. CNV is a form of variation, which affects the number of copies of a gene, resulting in increased or reduced protein expression. In the following sections, we give an overview of known and proposed α_2_δ disease associations. To this end, we distinguish between disease mutations in the proteins, which have been at least partially verified or discovered in specific patients and identified genetic disease associations for the human genes CACNA2D1–4, which partially lack experimental confirmation.

### Disease mutations in α_2_δ proteins

Here, we briefly summarize human α_2_δ protein mutations, which were identified in PubMed, ClinVar, and SFARI Gene databases and which can be traced back to at least one patient. However, until today, the functional consequences of these mutations were either not or only partially characterized.

#### α_2_δ-1

Missense or protein-disrupting mutations in α_2_δ-1 have mainly been found in epileptic disorders (Fig. 1, hα_2_δ-1). Two large genomic deletions affecting the genetic region of α_2_δ-1, a de novo mutation (8.2 Mb deletion) and an inherited mutation (3.9 Mb deletion), were identified in patients with epileptic encephalopathy [68]. The same study identified additional inherited disease mutations (p.Ser709Asn and p.Asp1045Ala) occurring in two families: family 1 with an unaffected mother and dizygotic twin having the same mutation and family 2 with an unaffected father and affected monozygotic twin having the same mutation. Another de novo mutation (p.Val875fs) was discovered in a West syndrome patient [44]. A whole exome sequencing (WES) study revealed a de novo mutation p.Arg351Thr in a family of the Simons Simplex Collection (SSC) of patients with autism spectrum disorders (ASDs) [51].

#### α_2_δ-2

Disease mutations in α_2_δ-2 have been identified in epileptic encephalopathy and cerebellar ataxia patients (Fig. 1, hα_2_δ-2). Edvardson et al. [34] described the p.Leu1040Pro mutation located in exon 36, close to the missense mutation p.Asp991Asn in exon 34 [115]. Another missense mutation (p.Pro261Leu) was identified in exon 7 [12]. Punetha et al. [84] found two families with epileptic encephalopathy: one displayed a missense variant mutation in exon 20 (p.Arg593Pro) while the other family exhibited a nonsense mutation in exon 5, predicting a severely truncated protein (p.Tyr162Ter). Furthermore, a CACNA2D2 variant (A900T) was found in schizophrenia (SCZ) patients within a Spanish population [92]. Finally, a frameshift mutation in exon 13 (p.Asn432fs) is likely causal in a family with epileptic encephalopathy [81].

#### α_2_δ-3

Potential disease-causing mutations in α_2_δ-3 were identified in patients with ASDs (Fig. 2, hα_2_δ-3). Exome sequencing identified several synaptic, transcriptional, and chromatin genes disrupted in ASD including the α_2_δ-3 mutations p.Arg578Gln, p.Arg665Cys, p.Ala698Thr, p.Arg731Gly, p.Asp756Asn, p.Arg205Gly, and p.Glu508Ter [28]. α_2_δ-3 de novo gene mutations were also found in a Chinese ASD cohort (p.Ala773Val and p.Ala275Thr) [121] and in probands from the autism clinical and genetic resources in China (p.Ala917Thr and p.Ala698Val) [42].

#### α_2_δ-4

α_2_δ-4 mutations are strongly involved in retinal disease and gliomas, which go beyond the scope of the current review. However, we would like to highlight two prominent mutations (Fig. 2, hα_2_δ-4), which affect α_2_δ-4 expression or structure and may, in theory, also affect the previously suggested expression of α_2_δ-4 in the brain [95, 117]. A mutation found in night blindness (p.Tyr802Ter) results in a premature stop at amino acid 802. However, it is expected that the mutated messenger RNA (mRNA) is recognized by the nonsense-mediated decay machinery [127]. Another mutation that deserves mentioning is p.Arg707His found to be involved in cone-rod dystrophy [4]. In addition, large genomic deletions affect α_2_δ-4 protein expression and thereby cause retinal degeneration and night blindness. So far, no brain-related phenotypes were reported, although the gene has been identified as a risk gene for psychiatric disorders. One deletion of 35,740 bp comprising amino acids 574–850 was found in patients with bipolar disorder (BPD) [116].

### Genetic disease associations

In this chapter, we briefly summarize human CACNA2D disease associations, which were identified in PubMed, ClinVar, and dbSNP databases. Mutations and SNPs that were not validated in separate publications were not included.

#### CACNA2D1

Over the past years, various genetic studies implied CACNA2D1, the gene encoding the α_2_δ-1 isoform, in various forms of epilepsy and psychiatric disorders including major depressive disorders (MDDs), BPD, and SCZ (Fig. 1, CADNA2D1). In GWAS, CACNA2D1 was identified as a potential drug target in MDD [49] and SNP rs17156280 was associated with an interaction between depressive states and stressful events [50]. Furthermore, two SNPs (rs258668 and rs258677) were linked to depressive traits such as subjective well-being and neuroticism [76]. GWAS analysis of data from the Bipolar Disorder Genome Study Consortium [125] identified SNP rs2367911 as a risk for BPD with comorbid binge eating. In a haplotype analysis on Han Chinese population, two SNPs (rs37076|rs37093 G-C and rs10265730|rs258665 G-A) were associated with SCZ [134]. Within a Swedish population, a disruptive (frameshift) variant in CACNA2D1 has been identified in an exome sequence of schizophrenia patients [85]. A large deletion of CACNA2D1 was found in one SCZ patient in a Japanese population [66]. By comparing epileptic patients with control subjects, 23 SNPs were identified, of which 6 occurred exclusively in affected individuals [55]. From these 6 SNPs, one was located in the coding sequence (exon 11), one in the 3′ UTR, while the other 4 SNPs were located in introns including SNP (rs2302384), which was already known from the dbSNP database. Importantly, chromosomal deletions affecting CACNA2D1 were identified in three patients with epilepsy and intellectual disability [118].

#### CACNA2D2

Three de novo variations in CACNA2D2 (Fig. 1, CACNA2D2) were found in patients with SCZ [85]. Two out of three CACNA2D2 variations introduced premature stop codons while the third variant was predicted to disrupt a splice donor site (Purcell A/C). Another CACNA2D2 SNP (rs11917269) has been associated with BPD [103].

#### CACNA2D3

Initially, the gene encoding for α_2_δ-3 was characterized as a target for pain treatment, since the SNP rs6777055 was associated with reduced thermal pain response [72]. Nevertheless, more recently, CACNA2D3 has been linked to neurodevelopmental disorders such as SCZ, BPD, MDD, nicotine dependence (ND), and especially to ASD [52] (Fig. 2, CACNA2D3). For example, the genetic region of CACNA2D3 (3p14) was associated with SCZ and an endophenotype related to the function of the temporal lobe, the anti-saccade reflex [63]. In another study, pathway analysis of SCZ risk SNPs suggested an association of CACNA2D3 with the response to lurasidone, an antipsychotic drug used to treat SCZ [62]. Furthermore, regional enrichment analysis associated the genomic region 3p21.1_1, which also contains CACNA2D3, with SCZ and BPD [63], a link which also is supported by other studies [63, 67, 70, 101, 134]. While the abovementioned SNPs are all located in exons, one intronic SNP (rs75407252) has been linked to MDD [33]. Associations with ASD were identified in whole genome sequencing studies as inherited variations resulting in splicing disruption [131] or as CNVs [41]. Moreover, SNP rs3773540 was among the top 15 SNPs contributing to ASD diagnosis [100] and a splice site mutation at the beginning of exon 24 was identified in an ASD patient [52]. In their exome sequencing study, De Rubeis et al. [28] further identified several inherited variations in CACNA2D3 with so far unknown effect. Finally, 30 CACNA2D3 SNPs were identified in a study on epilepsy, of which 6 occurred in epileptic patients [55].

#### CACNA2D4

α_2_δ-4 is almost exclusively expressed in the retina, pituitary gland, and adrenal gland [29, 88]. However, very low levels are also detected in the hippocampus and are upregulated during development and status epilepticus [95, 117]. A possible role in the brain, which is further outlined below, is suggested by several SNPs linked to numerous neurological disorders. For instance, a SNP located between the CACNA2D4 and CACNA1C genes (rs1024582) was significantly associated with cross-disorders that included attention deficit hyperactivity disorder (ADHD), BPD, ASD, SCZ, and MDD [22]. A de novo frameshift mutation likely disrupting CACNA2D4 was found in patients with SCZ [85] (Fig. 2, CACNA2D4). Another endophenotype of SCZ is default mode network, which was found to associate with the SNP rs4765847 [67]. SNPs rs2041140 and rs2041141 were linked to BPD [83], and partial deletions of 35.7 kb, eliminating exons 17–26, were found in two unrelated patients with late-onset BPD, one deletion was found in control individuals [116]. A WES study, comparing brain samples from MDD patients that died from suicide with MDD patients dying from unrelated causes, found a splice donor variant (C/A) [111]. Furthermore, a rare homozygous deletion affecting CACNA1C and CACNA2D4 (12p13.33) was found in a male ASD patient [102]. Thirty-nine SNPs were associated with epilepsy, of which 13 were exclusively found in the patients and not in controls [55] (Fig. 2, CACNA2D4).

## Potential disease mechanisms

The overall picture of identified disease mutations suggests the involvement of α_2_δ proteins in epilepsy (particularly α_2_δ-2 but also α_2_δ-1) and ASD (mainly α_2_δ-3 but also α_2_δ-1). The majority of identified mutations represent missense mutations resulting in single amino acid substitutions and, to a lesser extent, protein truncations resulting in the complete or partial loss of α_2_δ proteins (e.g. p.Tyr162Ter in α_2_δ-2 or p.Glu508Ter in α_2_δ-3). When considering genetic disease associations, the picture becomes more diverse and links α_2_δ genes additionally to MDD (α_2_δ-1, α_2_δ-2, and α_2_δ-3), SCZ (α_2_δ-1, α_2_δ-2, α_2_δ-3, and α_2_δ-4), BPD (α_2_δ-1, α_2_δ-2, and α_2_δ-4), and ND (α_2_δ-3) [129]. While some of the described SNPs are in exons and most likely result in missense mutations, many are found in intronic DNA stretches or are affecting splice sites. As α_2_δ proteins, on the one hand, are important modulators of calcium channels and, on the other hand, are independent regulators of synaptic functions, disease mutations can affect these functions either independently or concomitantly. In order to understand which cellular functions may be compromised by α_2_δ mutations, we will here recapitulate evidence for the involvement of α_2_δ proteins in calcium channel–dependent and calcium channel–independent functions.

### Calcium channel–dependent mechanisms

**Fig. 3:**
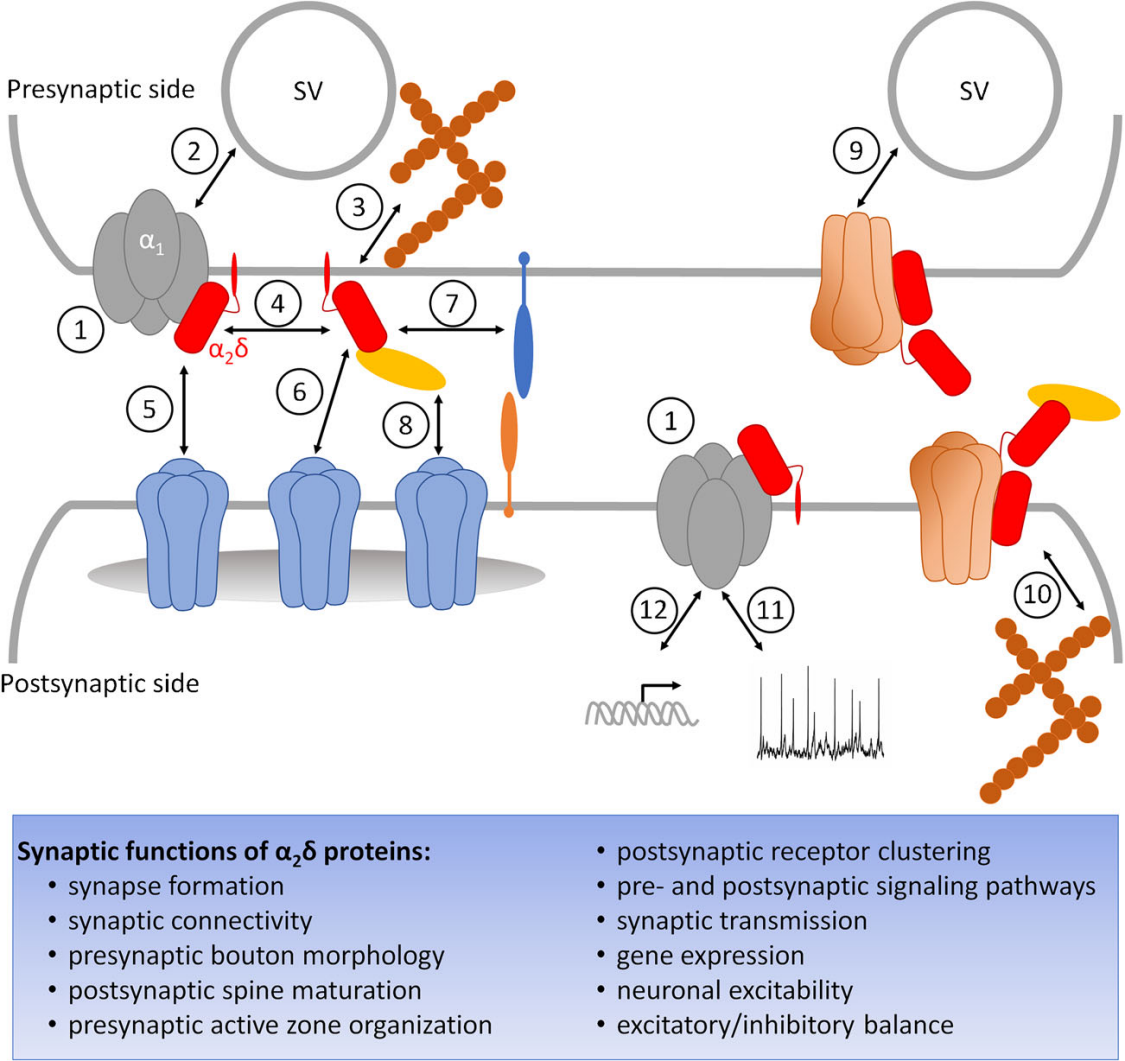
Model summarizing proposed synaptic functions of α_2_δ proteins. α_2_δ proteins as calcium channel subunits enhance plasma membrane expression and modulate current properties of both presynaptic and postsynaptic α_1_ subunits (1). At the presynaptic terminal, α_2_δ proteins mediate the accumulation of synaptic vesicles (SV, 2). They regulate active zone architecture (AZ) and bouton morphogenesis (3) either directly by interacting with proteins of the AZ and cytoskeleton (3) or indirectly via the VGCC complex (4). By aligning the presynaptic AZ with the postsynaptic membrane and postsynaptic AMPAR and GABA_A_R (receptors in blue; 5 and 6, respectively), they act as trans-synaptic organizers either partly (5) or entirely (6) independent of the VGCC complex. This may be mediated by a direct interaction with postsynaptic receptors (6), by interacting with prototypical cell adhesion molecules such as presynaptic neurexins and postsynaptic neuroligins (7), or by interacting with proteins of the extracellular matrix or secreted proteins (e.g. BDNF, TSP; yellow ellipses) (8). A transmembrane form of α_2_δ-1 bound to TSP is suggested to initiate the recruitment and stabilization of NMDAR (receptors in auburn) on the presynaptic (9) and postsynaptic (10) surface, a mechanism which contributes to enhanced synaptic transmission (9) and regulates intracellular signaling pathways as well as dendritic spine maturation via a small Rho GTPase (10). By regulating calcium currents of VGCC, α_2_δ proteins are further predicted to modulate neuronal excitability (11) and gene expression (12)

In heterologous co-expression studies, all α_2_δ proteins show similar effects on channel surface expression (reviewed in [25, 39, 57]) (Fig. 3, point 1). In contrast, evidence in differentiated native cellular systems suggests at least a partial isoform specificity. In rat superior cervical ganglion neurons, for example, recombinant expression of α_2_δ-1 and α_2_δ-2 differently affected Ca_V_2.2 expression when compared with α_2_δ-3 [98]. In mice, genetic ablation of α_2_δ-1 abolishes Ca_V_2.2 cell surface expression in dorsal root ganglion (DRG) neurons and dramatically reduces dorsal horn expression [73]. Deletion of α_2_δ-3 differentially affects calcium currents; it reduces P/Q- and R-type currents, while N- and L-type remain unaltered [104]. Loss of α_2_δ-3 blocks homeostatic modulation of neurotransmitter release due to a failure to potentiate presynaptic calcium influx [122].

Kinetic and voltage-dependent properties of the channels are also modulated by α_2_δ proteins: depending on the channel type and the native channel environment, activation and inactivation kinetics can be slowed or accelerated [74, 75, 114, 120]. The voltage dependence of steady-state inactivation is generally hyperpolarized by α_2_δ [36], while a shift to more positive potential is observed in individual cases [87]. In this context, it is noteworthy that the presence of α_2_δ-1 facilitates channel activation by increasing the voltage sensitivity of the channel’s voltage-sensing domains [93]. Another important structural property of α_2_δ which affects calcium currents includes the VWA domain, as a MIDAS mutant of α_2_δ-2 subunit failed to enhance Ca_V_1.2, Ca_V_2.1, and Ca_V_2.2 currents [14].

In neurons of the brain and the sensory nervous system, calcium channel function can also be influenced by altered or aberrant interaction of α_2_δ proteins with other identified interaction partners. For example, thrombospondin (TSP)-4 can increase calcium currents after low-voltage depolarization and contributes to aberrant excitatory synaptogenesis associated with neuropathic pain development; both of these effects can be blocked by gabapentin [77, 130]. The synaptic cell-adhesion molecules α-neurexins together with α_2_δ-1 regulate presynaptic calcium influx through Ca_V_2.1 channels [10] (Fig. 3, points 1, 4, and 7). The inhibitory effect of prion protein [99] may be modulated by competing with α_2_δ for GPI-anchoring pathways [1]. Moreover, low-density lipoprotein receptor–related protein 1 (LRP1) reduces α_2_δ-1 trafficking and hence influences trafficking of Ca_V_2.2 to the cell surface [53]. Another protein suggested to influence VGCC function by interacting with α_2_δ-1 is the α subunit (Slo1) of the large conductance calcium-activated potassium (BK) channel [133]. Following spinal nerve injury, Slo1 regulates excitability by preventing the association of α_2_δ-1 and α_1_ subunits, resulting in reduced functional membrane expression of Ca_V_2.2 and analgesia. Similar mechanisms to counteract neuronal hyper-excitability might also be relevant in pathologic conditions, for example in epilepsy.

It is worth mentioning that an α_2_δ-like protein, CACHD1, has recently been characterized [20]. CACHD1 causes a functional increase of low voltage–activated T-type (Ca_V_3) currents, which are not modulated by classical α_2_δ proteins. However, despite having a disrupted MIDAS motif, rat Cachd1 also can increase Ca_V_2.2 currents and surface expression upon heterologous co-expression [23].

Taken together, it is well established that α_2_δ proteins have significant effects on the biophysical properties of calcium channels, particularly on calcium current density, activation and inactivation kinetics, and voltage dependence of activation and inactivation. Thus, potential mutations affecting α_2_δ expression levels, glycosylation, or attachment of the GPI anchor may generally affect calcium channel surface expression and hence calcium currents. The p.Leu1040Pro mutation associated with epilepsy [34] resulted in reduced current densities and slowed inactivation when co-expressed with neuronal calcium channels. Similarly, a point mutation in the VWA domain, identified in a patient with autism (p.Arg351Thr) [51], prevents stable plasma membrane expression of α_2_δ-1 in a heterologous expression system [90]. Both mutations suggest that these effects can be mediated via altered expression of presynaptic and postsynaptic calcium channels or defective calcium currents which may, ultimately, result in an aberrant excitatory-inhibitory balance and underlie the pathophysiology of the associated diseases (Fig. 3). Interestingly, structure-homology modeling revealed two critical interactions of Arg351 (data not shown); hence, a significant effect of the exchange to Thr on the protein structure is possible. However, it is important to note that for the majority of identified human α_2_δ mutations, the structure-function consequences have not yet been studied.

### Channel-independent synaptic functions of potential relevance for neurological disorders

In this chapter, we summarize novel synaptic roles of α_2_δ proteins, which, alone or in combination with channel-dependent functions, may be casually involved in neurological disorders: synapse formation, synaptic connectivity and postsynaptic receptor abundance, presynaptic architecture, and synaptic transmission (Fig. 3). The link between synaptic α_2_δ proteins and brain disorders is particularly relevant, as a common feature of neurological disorders is their linkage to synaptic dysfunctions, referred to as synaptopathies (reviewed in [113]). This particularly accounts for neurodevelopmental disorders such as ASD, intellectual disability (ID), Fragile X syndrome (FXS), Down syndrome, ADHD, epilepsy, neuropsychiatric disorders (e.g. BPD, SCZ), MDD, and neurodegenerative diseases such as Alzheimer’s, Huntington’s, and Parkinson’s diseases. Correct assembly of glutamatergic and GABAergic synapses comprises a series of intricate steps, including the contact of presynaptic axons with specific cellular postsynaptic compartments of target cells, as well as the recruitment of presynaptic and postsynaptic proteins to form a functional synapse (reviewed in [21, 38, 61, 110]). Hence, aberrant expression of individual synaptic proteins as well as mutations affecting their function might cause perturbations in synapse physiology and morphology. This, in turn, could lead to synaptic dysfunction, an imbalance of synaptic excitation and inhibition (E/I imbalance), abnormalities in wiring of neuronal circuits, and finally, the development of a neurological disease.

#### α_2_δ-1 mediates excitatory synaptogenesis

The interaction of postsynaptic α_2_δ-1 and its astrocyte-secreted ligand TSP mediates excitatory synapse formation in retinal ganglion cells, and the study suggests that α_2_δ-1/TSP-induced synaptogenesis does require neither presence nor function of α_1_ subunits [35]. A follow-up study [90] demonstrated that loss of α_2_δ-1 in a conditional knockout mouse strain [78] impairs excitatory synaptogenesis and spine morphology in the cortex (Fig. 3, point 10). Interestingly, a point mutation in the VWA domain of α_2_δ-1, which has been linked to autism in humans [51], prevented both membrane trafficking of α_2_δ-1 (see above) and the rescue of the synaptogenic defects. These findings suggest that the previously identified α_2_δ-1 disease mutation might underlie impaired excitatory synapse formation and synaptic transmission. The study by Risher et al. [90] also proposes that TSP binding to a transmembrane form of α_2_δ-1 initiates the recruitment and stabilization of NMDA receptors (NMDARs) on the postsynaptic surface, a mechanism which may consequently contribute to dendritic spine maturation (Fig. 3, point 10). The idea that α_2_δ-1 may be also associated with presynaptic function is further suggested by studies addressing its involvement in neuropathic pain [18], hyperalgesia [30], and cultured hippocampal neurons [7]. Increased α_2_δ-1 expression levels via lentiviral injection [18] or induced by opioid application [30] potentiated presynaptic NMDAR trafficking in spinal cord synapses (Fig. 3, point 9). Excitatory synaptic transmission was enhanced, ultimately provoking pain hypersensitivity, which could be normalized with either gabapentin or an α_2_δ-1 interfering peptide. The causative interaction of α_2_δ-1 with a set of distinct NMDAR subtypes (GluN1, GluN2A, and GluN2B) is presumably specific, as neither α_2_δ-2 nor α_2_δ-3 co-immuno-precipitated with NMDARs in the spinal cord and HEK cells [18]. To unravel the NMDAR interaction site within the α_2_δ-1 sequence, a chimeric approach was used, substituting the C-terminus, α_2_, or δ peptide of α_2_δ-1 with that of α_2_δ-2 or α_2_δ-3. Surprisingly, the interaction domain was identified to be located after the C-terminal GPI anchor cleavage site. As earlier studies revealed that α_2_δ subunits have hardly any or no cytoplasmic domain [26], these data might corroborate the abovementioned findings that NMDARs bind to a transmembrane form of α_2_δ-1 (for further detail and discussion, see [32, 46]) (Fig. 3, points 9 and 10). Both the hypothalamus [64, 65] and striatum [136] express presynaptic and postsynaptic α_2_δ-1/NMDAR complexes, augmenting NMDAR-mediated synaptic glutamate release in disease (hypertension) [64, 65] and under physiological conditions (corticostriatal long-term potentiation in learning and memory) [136]. However, since a proteomic study of native rat brain [79] could not identify α_2_δ subunits in NMDAR-rich postsynaptic densities, it is conceivable that NMDAR/α_2_δ-1 association might represent a rather dynamic form of neuronal signaling primarily relevant in neuropathological conditions.

It has been recently suggested that brain-derived neurotrophic factor (BDNF) might be an upstream regulator of neuronal α_2_δ-1 expression levels [9, 19, 86]. Lack of BDNF in mice causes reduced α_2_δ-1 cell surface expression in the ventromedial hypothalamus, likely affecting excitatory synapse formation without obviously altering calcium currents [19]. Moreover, BDNF mutant mice recovering from stroke display increased abundance of α_2_δ-1 and TSP2 together with a concomitant enhancement of glutamatergic synapses within the cortico-striatal pathway [86]. Importantly, as the study further identified a reduction of GABAergic innervation in distinct cortical layers, these data might suggest that synaptic α_2_δ-1 expression levels regulate the balance of excitation to inhibition.

#### α_2_δ-2 splice variants regulate synaptic connectivity and postsynaptic receptor abundance

Recent evidence suggests that presynaptic α_2_δ-2 potently regulates synaptic connectivity and postsynaptic receptor abundance at specific central synapses. For instance, we previously found that solely α_2_δ splice variants lacking exon 23 regulate both the wiring of presynaptic axons to GABAergic postsynaptic sites, as well as postsynaptic GABA_A_ receptor (GABA_A_R) abundance [40] (Fig. 3, points 5 and 6). As heterologous co-expression of distinct α_2_δ-2 splice variants with various α_1_ subunits caused similar effects on calcium current densities and activation/inactivation kinetics [45], these data suggest that the trans-synaptic function of α_2_δ-2 variants lacking exon 23 is independent of their role as a calcium channel subunit. Interestingly, α_2_δ-2 is also necessary for the proper spatial alignment of presynaptic L-type calcium channels and postsynaptic AMPA receptors in hair cell synapses of the inner ear [37], as well as for the structure and function of cerebellar climbing fiber synapses [5]. While the distinct signaling pathways remain to be determined, it is thus tempting to speculate that glutamatergic and GABAergic synapses may express a specific set of presynaptic α_2_δ isoforms and even splice variants in order to regulate synaptic connectivity and postsynaptic receptor abundance during development (Fig. 3, points 5 and 6). Along these lines, previous studies have proposed developmental functions of an α_2_δ-2 variant lacking exon 23 in establishing neuronal circuits [106, 108, 109]. As murine axonal projections of DRG neurons mature during embryonic development, they undergo a switch from a growth-competent (electrically dormant) to a transmitting (electrically active) phase, which also correlates with increased α_2_δ-2 expression levels [108]. Whether α_2_δ-2 regulates axon growth of sensory neurons by altering α_1_ subunit–mediated neurotransmission needs to be clarified. However, recent findings in mouse cortical neurons show that α_2_δ-2, which is specifically expressed at the soma, axons, and growth cones of corticospinal layer V neurons, displays distinct postnatal expression patterns [106]. Therefore, increased α_2_δ-2 expression levels during development accompany increased spontaneous firing at a time point when cortico-spinal axon growth is nearly completed, and synaptogenesis begins.

Altogether, several lines of evidence suggest the existence of distinct spatiotemporal expression patterns of α_2_δ-2 regulating synapse connectivity and specificity, which may even depend on exon usage [40]. Since the amount of α_2_δ-2 protein is also increased in pathological conditions such as spinal cord injury [106, 108], abnormal expression levels likely contribute to maladaptive synaptogenesis or plasticity, ultimately leading to aberrant neuronal networks.

#### α_2_δ-3 regulates size, morphology, and architecture of presynaptic boutons

Most of today’s knowledge on the role of α_2_δ-3 in synaptic transmission and synapse formation is based on studies conducted in invertebrate model systems. Caylor et al. [16] showed, for instance, that in *Caenorhabditis elegans*, homologs of Ca_V_2 (UNC-2), α_2_δ (UNC-36), and CaMKII (UNC-43) regulate the size and morphology of GABAergic motoneuron terminals in neuromuscular junctions (NMJs) (Fig. 3, point 3). In *Drosophila melanogaster* null mutants of the α_2_δ-3 homolog straightjacket (stj), motoneuron terminals of NMJs fail to develop presynaptic boutons showing a severely disrupted cytoskeleton [59]. Nevertheless, growth cones successfully navigate and halt at their target muscles, indicating that initial contact formation of synapses is normal but followed by an arrest of morphogenesis during larval development. Although the synapse-stabilizing protein ankyrin-2 is absent, functional presynaptic specializations are present and properly opposed to postsynaptic clusters of glutamate receptors. The Ca_V_2 homolog cacophony, however, is missing in active zones, consistent with an inability to evoke synaptic release. Both bouton formation and function could be rescued when expressing an α_2_δ-3 mutant lacking the δ peptide, suggesting that the synaptogenic function and calcium channel targeting property require the extracellular α_2_ part of the protein. Importantly, the authors demonstrated that bouton formation does not depend on synaptic calcium channel localization, as the α_1_ mutant cacophony displays normal morphogenesis. A recent study might offer a mechanistic explanation how the extracellular α_2_ peptide of α_2_δ-3 regulates the development of embryonic *Drosophila* NMJs. Hoover et al. [47] uncovered that presynaptic α_2_δ-3 promotes the function of an activity-dependent autocrine bone morphogenetic protein (BMP) signaling pathway via modulating membrane retention of glass bottom boat (Gbb). Presynaptic Gbb, in turn, serves as a retrograde cue regulating active zone architecture, synaptic vesicle distribution, neurotransmitter release, and bouton morphogenesis. Similar mechanisms might also exist in vertebrate synapse formation, as altered expression of mammalian α_2_δ-3 has been shown to affect the size and morphology of presynaptic boutons of auditory nerve fibers [82] (Fig. 3, point 3), and GABAergic synapses of central neurons as well as postsynaptic receptor abundance [40] (Fig. 3, points 5 and 6). In hippocampal neurons, overexpression of α_2_δ-3, in contrast to α_2_δ-1, facilitated spontaneous GABA release and increased the density of inhibitory synapses [7]. It is important to note that presynaptic α_2_δ-3 and α_2_δ-2 may regulate synaptic differentiation and postsynaptic receptor abundance by two independent mechanisms: while overexpression of α_2_δ-3 induced smaller synapses, associated with reduced content of both presynaptic and postsynaptic proteins, presynaptic α_2_δ-2 modulated postsynaptic GABA_A_R abundance without affecting presynaptic bouton size [40].

#### α_2_δ-4 is required for synaptic transmission and wiring of photoreceptors

α_2_δ-4 transcript levels by far exceed those of other α_2_δ isoforms in retinal photoreceptor cells [58]. Accordingly, mutations in the human, mouse, and zebrafish genes [54, 94, 123, 127, 128] and the associated pathological phenotypes underline a general importance of α_2_δ-4 in maintaining proper rod and cone synaptogenesis and physiology. Because α_2_δ-4 regulates both functional membrane expression of Ca_V_1.4 channels and synaptic transmission of rods [123] and cones [54, 94], converging lines of evidence suggest that abnormal Ca_V_1.4 expression may be the main cause for synaptic abnormalities of retinal photoreceptor cells. Nevertheless, these studies also implicate the presence of distinct α_2_δ-4 signaling pathways in rod and cone photoreceptors. For instance, Wang et al. [123] showed that in α_2_δ-4 knockout mice, the key synaptogenic molecule for rod synaptogenesis (Elfn1) is not recruited to rod synaptic terminals. This prevents rods from establishing contacts with their postsynaptic targets, the ON rod bipolar cells, which disrupt postsynaptic metabotropic glutamate receptor 6 (mGLUR6) clustering. Even though only little structural effects have been reported for cone photoreceptor synapses in this study, synaptic transmission appears to be severely impaired also in cones. A second report using a different α_2_δ-4 knockout mouse [54] could further extend these findings in revealing that abnormal wiring of cone synapses is associated with impaired cone transmission through ON and OFF bipolar pathways. Nevertheless, abnormalities of ribbon synapses are more severe and Ca_V_1.4 channels are lost faster in terminals of rods than in cones. Because Elfn1 is not expressed in cone synapses [15], the findings described in both reports [54, 123] indicate that loss of Ca_V_1.4 may be the primary cause for synaptic abnormalities in rods and cones. Alternatively, it is conceivable that α_2_δ-4 regulates rod and cone synapse structure by two distinct mechanisms (one dependent on Elfn1 and one independent from Elfn1). A recent study conducted in zebrafish underpinned a functional divergence and different developmental expression patterns of two α_2_δ-4 variants in cone photoreceptors [94]. Interestingly, solely the loss of one variant, Cacna2d4b, specifically leads to the occurrence of mislocalized synapses during larval development. Thus, it is tempting to speculate that spatiotemporal expression patterns of distinct α_2_δ-4 variants might regulate synapse connectivity and specificity of rod photoreceptors.

Until recently, protein and mRNA expression levels of human and murine α_2_δ-4 seemed negligible in all previously examined CNS regions [40, 88, 95]. A recent study, however, reported increased levels of α_2_δ-4 mRNA in human hippocampal biopsies obtained from epileptic patients, an interesting finding in spite of the lack of information on absolute mRNA levels [117]. Considering that standard curve–based qRT-PCR studies from our group identified very low amounts of α_2_δ-4 mRNA in the mouse brain [40, 95], the recent findings suggest the existence of a subpopulation of hippocampal neurons expressing α_2_δ-4. Interestingly, several studies provide a potential link between CACNA2D4 and psychiatric disorders ([116], see discussion therein and above). Yet, it remains to be addressed whether late-onset bipolar disorder might be a secondary effect caused by visual impairments in these patients, or if α_2_δ-4 might play a more prominent role in central neurons of the brain.

### How do mutations affect α_2_δ protein function?

**Fig. 4:**
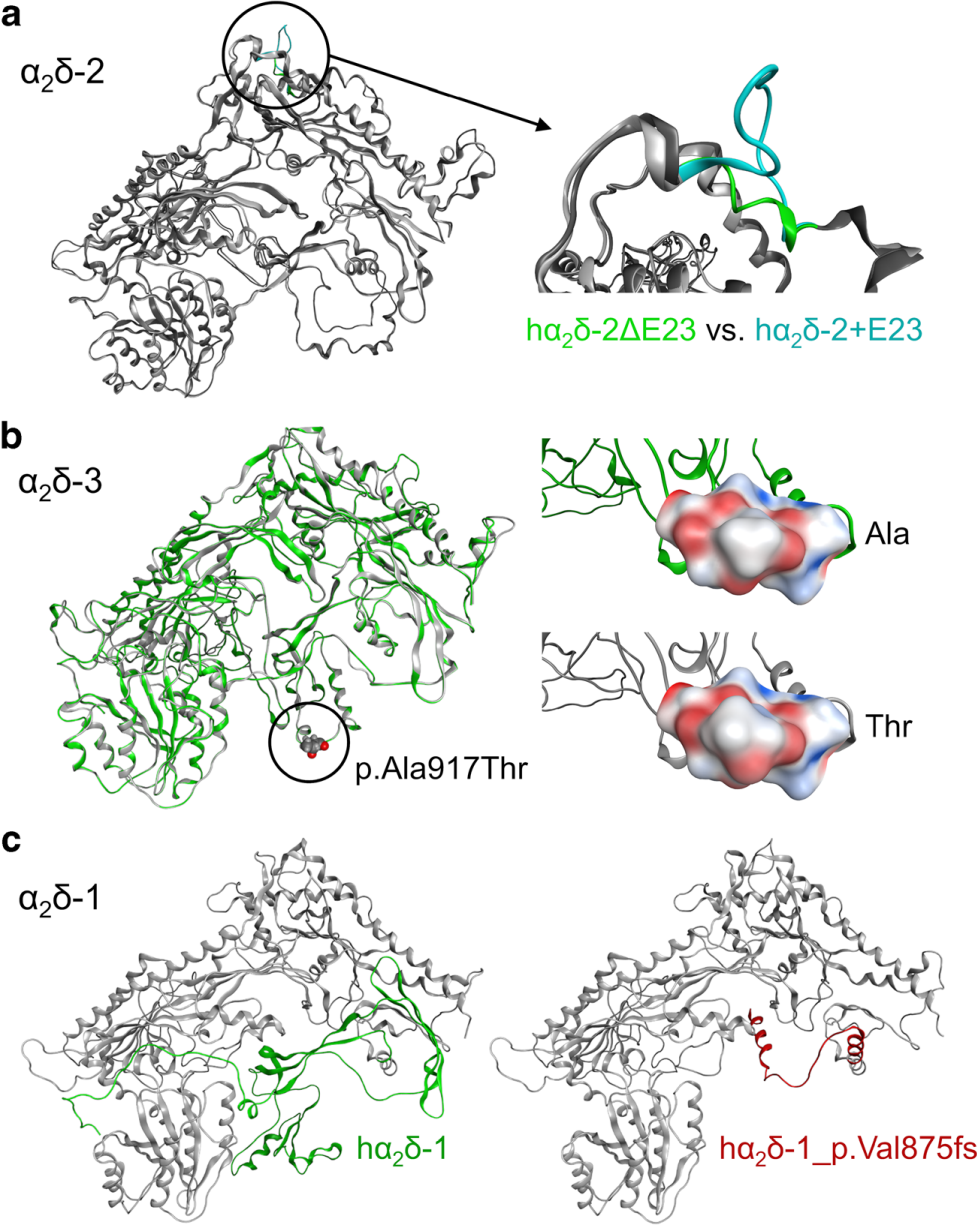
Putative consequences of α_2_δ splicing and selected disease mutations on protein structure. Using homology modeling based on the 2.7 Å resolution structure of α_2_δ-1 (PDB code: 6JP8) [6, 135], we tested the potential consequences of alternative splicing of exon 23 on the structure prediction of hα_2_δ-2 (**a**) [40], the potentially autism-causing mutation p.Ala917Thr in hα_2_δ-3 (**b**) [42], and the hα_2_δ-1 frameshift mutation p.Val875fs associated with epilepsy (**c**) [44]. **a** As previously suggested for mouse α_2_δ-2 [40], the inclusion of exon 23 in hα_2_δ-2 suggests the formation of an extra loop leading to the disruption of an α-helix present. **b** Mutation p.Ala917Thr is not predicted to alter hα_2_δ-3 protein structure; however, altered electrostatic potential (EP) on the surface (red, negative EP; blue, positive EP) and hence altered surface hydrophobicity may influence protein interactions. **c** The hα_2_δ-1 frameshift mutation p.Val875fs deletes the C-terminal part of α_2_ and the entire δ peptide (green). Furthermore, the altered reading frame introduces a random 36-amino acid sequence stretch (red). Molecular modeling was performed with the MOE software [69]. EP calculations were performed using Amber 10:EHT charges and the Poisson-Boltzmann approach as implemented in the software. Prior to the calculations, the structure was prepared and protonated with Protonate3D within MOE

Considering the manifold roles involving α_2_δ proteins, it is obvious that α_2_δ malfunction can affect channel-dependent and channel-independent functions. Particularly, mutations affecting expression levels of α_2_δ proteins (e.g. intronic SNPs, protein truncations, CNV) may simultaneously affect both functions. Hence, the extent of the disease involvement will mainly depend on the regional and temporal expression of individual α_2_δ subunits. For example, this is the case in epileptic encephalopathies and cerebellar ataxias associated with α_2_δ-2 (predominant expression of α_2_δ-2 in the cerebellum) or retinal dysfunctions associated with α_2_δ-4 (predominant expression of α_2_δ-4 in the retina). Similarly, mutations affecting splicing may primarily affect regions endogenously expressing specific splice isoforms. Although until today regional and temporal expression patterns of α_2_δ splice variants are still incompletely understood, splices are indeed relevant for the functional diversity [40, 60]. However, in contrast to mutations affecting α_2_δ protein expression levels, splicing mutations may affect channel-dependent and channel-independent functions simultaneously or separately. Homology modeling based on an α_2_δ-1 cryo-EM structure [126, 135] revealed that inclusion of single spliced exon 23 in α_2_δ-2 resulted in the formation of an extra loop disrupting an α-helix [40] (Fig. 4a). Hence, inclusion of this exon prevents the trans-synaptic recruitment of postsynaptic GABA_A_ receptors. Since, based on the predicted structure, the region of the relevant exon is facing away from the channel into the synaptic cleft, it is conceivable that such structural alterations affect specific protein-protein interactions without modulating the calcium channel. Channel-independent structural interactions are supported by a very recent observation in our laboratory. While the integrity of the MIDAS motif in α_2_δ subunits was shown to be required for functional membrane expression of the channel complex [14, 48, 97], expression of MIDAS mutants could not rescue the accumulation of synaptic proteins in α_2_δ subunit triple knockout neurons [97].

The consequences of missense point mutations on channel-dependent and channel-independent functions are not that easily predictable without detailed experimental evidence. In theory, missense mutations may alter the entire structure of α_2_δ proteins. Moreover, single missense mutations may also affect the interaction of α_2_δ subunits with the channel itself or with extracellular or trans-synaptic interaction partners. Here, we used structure-homology modeling (see Fig. 4) to predict the consequences of previously identified potentially autism-causing mutations in α_2_δ-1 and α_2_δ-3. While the p.Arg351Thr mutation may severely alter protein structure (see above), modeling of a specific mutation in α_2_δ-3 (p.Ala917Thr) [42] provides an indication that even subtle changes may alter specific interactions. The location of p.Ala917Thr in the predicted structure of α_2_δ-3 is a potential site for protein-protein interaction, partly facing towards the channel α_1_ subunit and partly facing to the extracellular side (Fig. 4b). Homology modeling does not suggest strong effects of this amino acid substitution on the overall structure. However, slight alterations in the surface electrostatic potential and hence surface hydrophobicity may suffice to influence the stability with specific inter-channel-complex interactions or components of the extracellular matrix. Considering ASD, one may speculate that an altered surface structure may influence the weak and dynamic interaction with synaptic cell adhesion molecules, for example neurexins [10], thereby affecting both channel and synaptic functions. We also modeled the potential consequences of an α_2_δ-1 frameshift mutation likely causing epilepsy (p.Val875fs) [44]. While it is possible that altered mRNA may already trigger nonsense-mediated mRNA decay, an actually translated α_2_δ-1_p.Val875fs protein may be functionally affected in two ways: first, the lack of the δ peptide and membrane anchoring will affect the stability of its membrane localization and hence channel interaction, and second, the frameshift not only deletes a large portion of the C-terminal end of the protein including the entire δ peptide but also introduces a sequence stretch of 36 amino acids which will likely interfere with potential remaining protein-protein interactions (Fig. 4c). Hence and considering that the p.Val875fs mutation was found in epileptic patients, it seems consistent with the hypothesis that its pathophysiology may be mediated partly by channel-dependent functions influencing neuronal excitability and/or synaptic transmission.

## Future perspectives

**Fig. 5:**
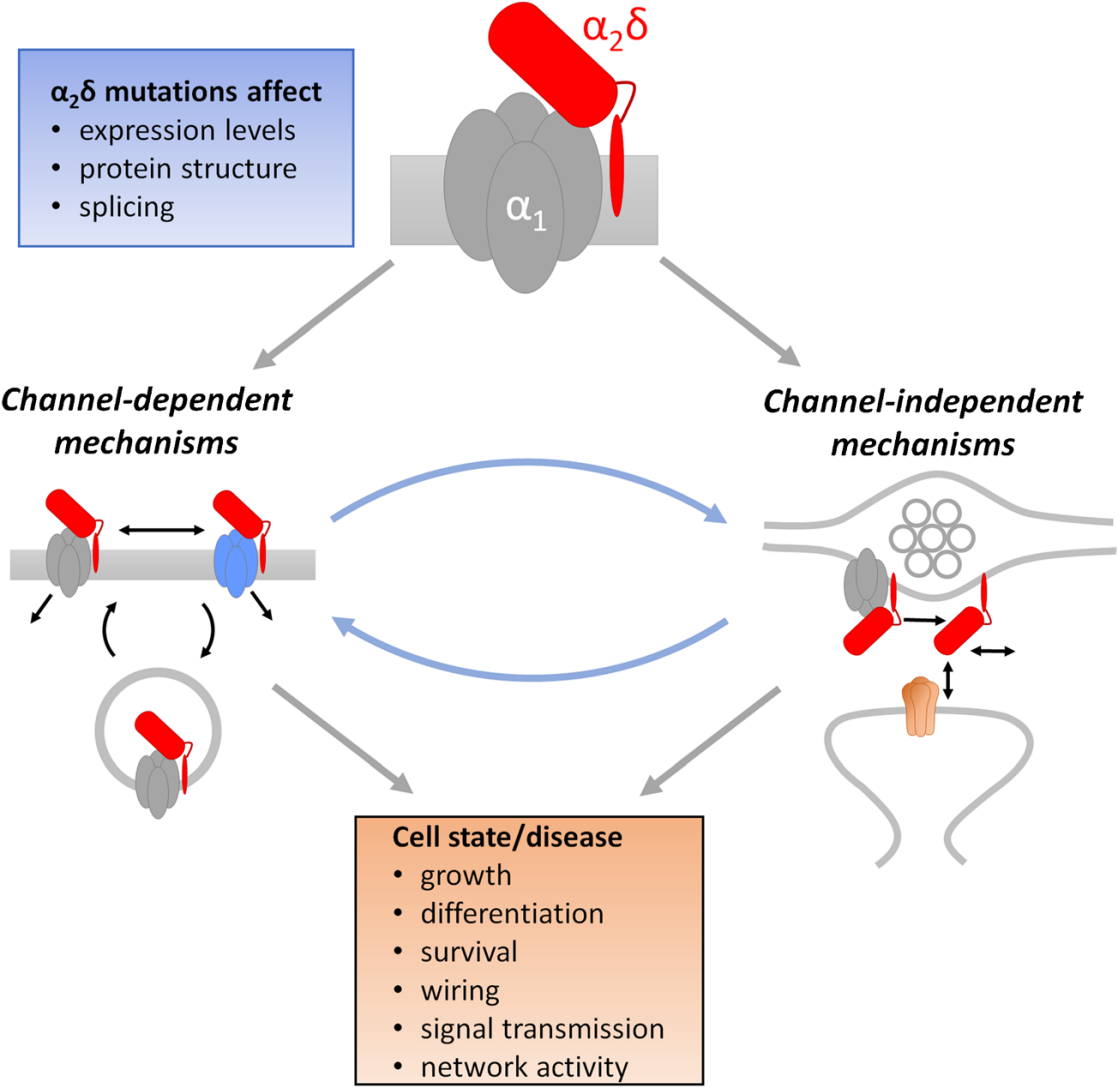
Model summarizing channel-dependent and channel-independent roles of α_2_δ proteins. Mutations of α_2_δ genes can affect expression levels, protein structure, and splicing. These alterations have consequences on calcium channel–dependent functions (membrane expression, current modulation, channel subtype–specific functions), and channel-independent functions (extracellular and/or trans-synaptic interactions and signaling pathways). The two mechanisms should not be considered entirely independent as they are likely influencing each other. For example, alterations in synapse differentiation will also affect VGCC expression with further consequences on neuronal excitability and synaptic transmission

Over the last couple of years, the number of disease associations of α_2_δ genes and proteins has been steadily increasing. The multitude of distinct neurological and neuropsychiatric disorders likely involving α_2_δ proteins supports their important roles in neuronal functions (Fig. 5). Hence, on top of the existing drugs gabapentin and pregabalin, α_2_δ proteins may provide highly specific targets for future and novel paradigms in treating neurological disorders. This seems particularly promising when calcium channel–dependent as well as calcium channel–independent functions could be separately targeted. However, until then, several basic immanent research questions need to be further elucidated: first, although recent studies identified α_2_δ isoform–specific signaling pathways, the role of functional redundancy between the different isoforms is not yet understood. For example, while specific α_2_δ isoforms are clearly associated with distinct synaptic functions (see above), synapse deficiency in a presynaptic triple knockout phenotype can be rescued by the expression of each individual α_2_δ protein [97]. Second, the role of individual α_2_δ isoforms in neuron-type- and synapse-type-specific signaling mechanisms needs to be resolved. In the brain, α_2_δ proteins show an isoform-specific distribution pattern; however, this pattern shows considerable overlap and at least three isoforms can be simultaneously expressed in a single neuron. Hence, understanding neuron- and synapse-type specificity in α_2_δ protein functions will become increasingly important. For example, the extremely low abundance of α_2_δ-4 in the brain may suggest the specific expression of this isoform in a lowly abundant neuronal cell type. Third, the definitive distinction between calcium channel–dependent and calcium channel–independent functions is until today an experimental challenge. Several recent studies identified α_2_δ-specific signaling pathways and protein-protein interactions (e.g. BK channel and NMDAR). Yet, in neurons, multiple calcium channel types are ubiquitously expressed and critically involved in basic signaling functions. Consequentially, malfunctioning α_2_δ proteins are likely to also affect calcium channel functions. Fourth, α_2_δ proteins are emerging as novel and critical trans-synaptic organizing molecules, which may provide a missing link in understanding synapse formation and differentiation. It will be critical to identify their specific trans-synaptic mode of action including potential extracellular or presynaptic and postsynaptic interaction partners. Finally, cryo-EM analysis provided first insights into the structural organization of α_2_δ subunits, but until today, our understanding of neuronal α_2_δ proteins depends on structure-homology modeling based on the skeletal muscle calcium channel complex. In neurons, the actual structural organization of α_2_δ proteins may depend on their specific calcium channel associations and their potential extracellular and trans-synaptic interactions.

Taking together, α_2_δ proteins are critical neuronal signaling proteins involved in a variety of cellular and synaptic mechanisms and altered function may cause or mediate neurological disorders. Future research efforts in studying their specific neuronal roles will help in understanding the pathophysiology of associated disorders and may open the way for the development of novel therapeutic paradigms.
